# Metabolic Memory-Mediated Epigenetic Regulation of EMT in Diabetic Kidney Disease: Mechanisms and Therapeutic Implications

**DOI:** 10.3390/ijms27114801

**Published:** 2026-05-26

**Authors:** Xinning Ran, Yidan Xu, Ruonan Liang, Yuqi Duan, Wanying Jia, Yuhong Bian, Chenduo Li, Mingxing Zhang

**Affiliations:** School of Integrative Medicine, Tianjin University of Traditional Chinese Medicine, Tianjin 301617, China; rxn971123@gmail.com (X.R.);

**Keywords:** DKD, renal fibrosis, epithelial–mesenchymal transition, endothelial–mesenchymal transition, metabolic memory, epigenetic mechanism, treatment strategies

## Abstract

Diabetic kidney disease (DKD) is a leading cause of end-stage renal disease, with renal fibrosis as its core pathological hallmark. A central driver of this fibrosis is epithelial–mesenchymal transition (EMT), during which renal tubular epithelial cells transform into matrix-producing myofibroblasts. Endothelial–mesenchymal transition (EndMT) has also emerged as a critical contributor, and together with EMT, accounts for the progressive accumulation of myofibroblasts and extracellular matrix. A major clinical challenge in halting DKD progression is “metabolic memory”, a phenomenon whereby renal injury persists and EMT/EndMT remain activated even after glycemic control is achieved. The molecular basis underlying this sustained activation remains incompletely understood. Emerging evidence indicates that metabolic memory is largely mediated by epigenetic mechanisms, including histone modifications, DNA methylation, and non-coding RNA dysregulation. These stable epigenetic imprints maintain the persistent activation of key pro-fibrotic signaling pathways, especially TGF-β, thereby continuously driving EMT, EndMT, and excessive extracellular matrix deposition. Although targeting epigenetic regulators has shown promising anti-fibrotic effects, a systematic review that integrates how metabolic memory orchestrates both EMT and EndMT through a multi-layered epigenetic network remains lacking. This review comprehensively summarizes the epigenetic mechanisms by which metabolic memory sustains EMT and EndMT in DKD, highlights key therapeutic targets, and discusses their translational and clinical implications.

## 1. Introduction

Diabetic kidney disease (DKD) is one of the most common and severe microvascular complications of diabetes, affecting up to 40% of diabetic patients and representing the leading cause of end-stage renal disease (ESRD) [1,2]. With the escalating global prevalence of diabetes, DKD has become a major public health challenge.

Despite advances in glycemic control and renoprotective therapeutics, the progression of DKD is still poorly controlled, with glomerulosclerosis and renal interstitial fibrosis as its core pathological features [3,4]. Renal fibrosis, characterized by abnormal deposition of extracellular matrix (ECM) and structural destruction of the kidney, commonly results in the development of ESRD. The accumulation of myofibroblasts from diverse origins plays a crucial role in mediating this process [5,6,7]. Among these, epithelial–mesenchymal transition (EMT) is well-accepted as a critical mechanism [8]. Phenotypic reprogramming is triggered in renal tubular epithelial cells in response to stimuli including hyperglycemia, inflammation, and oxidative stress, leading to losing epithelial markers and acquiring mesenchymal features, thereby promoting fibrosis. In addition, the contribution of endothelial–mesenchymal transition (EndMT) to renal fibrosis has been increasingly acknowledged, though it remains relatively less explored. During the process of EndMT, endothelial cells acquire a mesenchymal, fibroblast-like phenotype, instead of endothelial characteristics, directly driving microvascular rarefaction, tissue hypoxia, and ECM deposition. Together, EMT and EndMT constitute two important cellular pathways fueling the progression of renal fibrosis in DKD [9,10].

Notably, the concept of “metabolic memory” has been established based on a range of clinical and experimental studies, which describes the persistent molecular damage caused by prior hyperglycemic exposure that continues to drive disease progression even after subsequent glycemic normalization [11,12]. This phenomenon provides a compelling explanation for the unrelenting nature of DKD, as both EMT and EndMT remain sustained long after glucose control is achieved. The molecular basis of metabolic memory has long remained elusive, but recent research has revealed that epigenetic regulation plays a central role. Multi-level regulatory mechanisms, including DNA methylation, histone modification, and non-coding RNAs (ncRNAs), can stably alter gene expression patterns without changing the DNA sequence, thereby forming “epigenetic imprints” that durably affect cellular function [13].

In DKD, these epigenetic changes mediate the sustained activation of EMT and EndMT by regulating key signaling pathways, particularly transforming growth factor-β (TGF-β), thereby promoting renal fibrosis. Moreover, complex cross-regulatory networks exist among different epigenetic mechanisms; for instance, microRNAs (miRNAs) can target DNA methyltransferases and histone-modifying enzymes, while long non-coding RNAs (lncRNAs) and circular RNAs (circRNAs) regulate the expression of core genes involved in EMT and EndMT through competitive endogenous RNA (ceRNA) mechanisms, amplifying fibrotic signals.

Previous studies have examined EMT and epigenetic modifications in DKD from different perspectives. However, a systematic elucidation of how metabolic memory integrates and regulates EMT and EndMT through a multi-level epigenetic network remains lacking. Driven by emerging technologies such as single-cell RNA sequencing and spatial transcriptomics, the heterogeneity of kidney cells during fibrosis is increasingly recognized, and traditional EMT theory faces new challenges. Additionally, the interplay between metabolic reprogramming (e.g., enhanced glycolysis and lactate accumulation) and epigenetic modifications (e.g., histone lactylation) offers new research directions for understanding metabolic memory.

Based on the above background, this review systematically examines how metabolic memory sustains the activation of EMT and EndMT through epigenetic mechanisms in DKD renal fibrosis. It focuses on the molecular regulatory network of EMT and further analyzes the epigenetic basis of metabolic memory. The roles of DNA methylation, histone modification, and non-coding RNA in regulating EMT and EndMT are summarized, and potential therapeutic targets and their clinical translational prospects are explored, aiming to provide a theoretical basis for precise intervention strategies for DKD.

## 2. The Core Regulatory Mechanisms of Mesenchymal Transition in DKD

The progression of DKD is highly dependent on the persistent hyperglycemic microenvironment. In this context, upstream pathogenic factors, including the accumulation of advanced glycation end products (AGEs), exacerbation of oxidative stress, and activation of the renin–angiotensin–aldosterone system (RAAS), collectively drive the initiation and development of kidney injury [14]. These factors are interwoven through a complex signaling network that converges on two core downstream axes: the inflammatory signaling axis and the fibrotic signaling axis.

**Figure 1:**
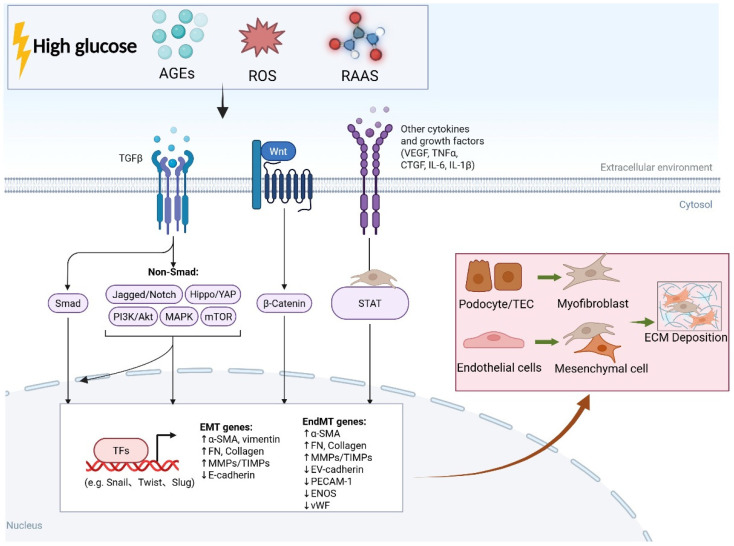
Hyperglycemia-induced AGEs, ROS, and RAAS activate key pathways including TGF-β (Smad and non-Smad), Wnt/β-catenin, and cytokine/STAT signaling, respectively. These pathways regulate transcription factors (e.g., Snail, Twist, Slug), driving EMT and EndMT gene reprogramming. Consequently, podocytes, tubular epithelial, and endothelial cells acquire mesenchymal phenotypes, leading to myofibroblast formation and excessive ECM deposition. Persistent activation of these pathways is a hallmark of metabolic memory-associated epigenetic regulation. “↑” increase or activation; “↓” decrease or inhibition. (Created in BioRender. Ran, R. (2026) https://BioRender.com/g3helok, accessed on 1 April 2026).

The activation of inflammatory pathways like nuclear factor kappa B (NF-κB) and JAK/STAT mediated by cytokines such as interleukin-6 (IL-6) and tumor necrosis factor alpha (TNF-α) results in the maintenance of a chronic inflammatory microenvironment [15,16,17,18]. Concurrently, the TGF-β signaling pathway, as a core driver of fibrotic remodeling, plays a crucial role in EMT and EndMT (Figure 1) [19,20,21].

### 2.1. TGF-β Signaling Pathway: The Central Hub of EMT

TGF-β is well-accepted as a key effector molecule in inducing EMT and plays a central regulatory role in DKD-related renal fibrosis. As a pro-fibrotic signaling hub, TGF-β directly drives ECM synthesis and deposition, and promotes renal structural remodeling by regulating the phenotypic transformation of multiple cell types.

At the molecular level, TGF-β signaling operates through canonical Smad-dependent pathways and non-canonical Smad-independent pathways, which are highly integrated through multi-level crosstalk to jointly regulate the fibrotic processes, especially EMT. Functionally, TGF-β exerts pro-fibrotic effects by upregulating downstream effectors like connective tissue growth factor (CTGF), which in turn promotes the synthesis and deposition of ECM components such as fibronectin and type I, III, and IV collagen [17,22]. Crucially, TGF-β directly induces phenotypic reprogramming of glomerular podocytes and tubular epithelial cells, thereby triggering the EMT process [16,22,23]. In podocytes, TGF-β-induced EMT is accompanied by downregulation of slit diaphragm proteins like nephrin and ZO-1, and upregulation of mesenchymal markers such as α-smooth muscle actin (α-SMA) and vimentin, impairing the filtration barrier. Additionally, the transformation of renal tubular epithelial cells into a myofibroblast-like phenotype is mediated by TGF-β by inducing the expression of EMT-related genes such as N-cadherin and vimentin, which leads to exacerbation of interstitial fibrosis as these cells secrete large amounts of ECM [10,24].

#### 2.1.1. Smad-Dependent Pathway

In the canonical pathway, TGF-β binds to type II receptors, recruiting and activating type I receptors, which phosphorylate receptor-regulated Smad (Smad2/3). Activated Smad2/3 forms a complex with Smad4 and translocates to the nucleus [8], directly regulating the expression of key EMT transcription factors such as Snail, Slug, and Twist [25,26,27,28]. Simultaneously, Smad signaling suppresses epithelial markers such as E-cadherin and promotes expression of mesenchymal markers such as α-SMA, fibronectin, and type I collagen [29,30]. This coordinated transcriptional regulation ultimately drives the conversion of epithelial cells to mesenchymal phenotypes.

#### 2.1.2. Smad-Independent Pathway

Beyond the canonical pathway, TGF-β activates non-Smad signaling cascades, including ERK/MAPK, p38 MAPK, and PI3K/AKT pathways [31,32,33]. Not only do these pathways independently regulate transcription of EMT-related genes, but also interact with Smad signaling to amplify fibrotic responses. For instance, the ERK/MAPK pathway can enhance nuclear translocation and transcriptional activity by phosphorylating the linker region of Smad2/3 [34,35]. The PI3K/AKT pathway acts as a key downstream effector of TGF-β; pharmacological inhibition of PI3K/AKT can block TGF-β-induced Snail activation, thereby inhibiting the EMT response [36,37]. Furthermore, this pathway can also inhibit NF-κB signaling, reducing the release of pro-inflammatory factors such as TNF-α and IL-6, highlighting complex crosstalk between inflammation and fibrosis [31].

### 2.2. Synergistic Effects of Other Pro-Fibrotic Signaling Pathways

The pro-fibrotic effect of TGF-β does not exist in isolation, but rather operates through extensive crosstalk with multiple signaling pathways, forming a multi-level regulatory network that further amplifies the EMT response.

#### 2.2.1. Notch Signaling Pathway

The Notch signaling is usually silent state in mature renal tissue, but it is abnormally activated in pathological conditions and participates in the process of renal fibrosis [38]. A close cross-regulatory relationship has been demonstrated between Notch and TGF-β signaling. On one hand, the Notch pathway, as an important downstream mediator of TGF-β1-induced EMT, can promote its transcription by directly binding to the promoter region of Snail1, thereby driving the activation of the EMT program [27]; on the other hand, recent studies have found that TGF-β1 itself can activate transcription factor Nrf2 by inducing NADPH oxidase 4 (NOX4) to trigger the generation of reactive oxygen species (ROS). As a result of translocation to the nucleus, activated Nrf2 can bind to the antioxidant response element (ARE) in the promoter region of the *Notch4* gene, thereby upregulating the transcription level of *Notch4*.

The activation of the Notch pathway not only directly induces the expression of the key transcription factor Snail in EMT, but its ligand Jagged1 and downstream target gene Hey1 can also serve as positive feedback regulators of this pathway, thereby constructing a self-amplifying signaling loop that continuously enhances pro-fibrotic signals and accelerates the renal fibrosis process [39]. Notably, this positive feedback regulatory network may play a key role in maintaining sustained activation of EMT and the “metabolic memory” effect.

#### 2.2.2. Wnt/β-Catenin Signaling Pathway

The Wnt/β-catenin signaling pathway is also considered a key regulatory axis in the initiation and progression of renal fibrosis. Pathological conditions such as high glucose result in the abnormal maintenance of cytoplasmic β-catenin and its nuclear translocation. It complexes with T cell factor/lymphoid enhancer factor (TCF/LEF) transcription factors in the nucleus, thereby activating the transcription of multiple target genes [40]. Furthermore, the Wnt/β-catenin signaling pathway can interact with the TGF-β/Smad signaling pathway, synergistically enhancing the expression of EMT-related genes, consequently promoting the transformation of renal tubular epithelial cells into mesenchymal phenotype [40,41].

At the molecular level, Wnt ligands (such as Wnt3a) bind to Frizzled receptors and low-density lipoprotein receptor-related protein 5/6 (LRP5/6) co-receptors on the cell membrane surface, forming receptor complexes that inhibit the activity of the β-catenin degradation complex composed of Axin, adenomatous polyposis coli (APC), and glycogen synthase kinase-3β (GSK-3β) [40]. This process prevents the phosphorylation and ubiquitination degradation of β-catenin, allowing it to accumulate in the cytoplasm and translocate to nucleus, ultimately initiating the transcription program of downstream target genes [40].

The continuously activated Wnt/β-catenin signaling pathway can significantly upregulate the expression of various fibrosis-related genes, including fibronectin, collagen I, and α-SMA, leading to the promotion of fibroblast activation and excessive deposition of extracellular matrix (ECM), creating a positive feedback amplification loop and accelerating the progression of renal tubulointerstitial fibrosis [42]. Therefore, pathological activation of the Wnt/β-catenin signaling pathway is considered one of the most important molecular mechanisms underlying renal fibrosis, and targeting of Wnt ligands or β-catenin may become a potential therapeutic strategy for intervening in renal fibrosis.

#### 2.2.3. Hippo-YAP/TAZ Signaling Pathway

Recent studies have revealed that Hippo pathway effectors YAP and TAZ play important roles in renal fibrosis, linking tissue mechanical changes to sustained EMT activation. Mechanical stress and increased ECM stiffness can activate YAP/TAZ, causing nuclear translocation and synergistic promotion of pro-fibrotic gene expression with Smad complexes [43,44]. This pathway illustrates how the fibrotic microenvironment itself can perpetuate signaling, a concept that aligns with the self-sustaining nature of metabolic memory.

### 2.3. The Role of EndMT in DKD

In addition to EMT of tubular epithelial cells, EndMT has been recognized as a crucial and previously underestimated mechanism driving renal fibrosis in DKD. EndMT is a process of phenotypic reprogramming where endothelial cells lose their endothelial characteristics and acquire a mesenchymal, fibroblast-like phenotype, contributing significantly to the pool of activated myofibroblasts [45]. This process is central to microvascular dysfunction and rarefaction, further exacerbating tissue hypoxia and amplifying the pro-fibrotic response.

At the cellular level, EndMT is characterized by the downregulation of endothelial markers such as vascular endothelial cadherin (VE-cadherin), CD31 (PECAM-1), and endothelial nitric oxide synthase (eNOS), alongside the upregulation of mesenchymal markers, including α-SMA, fibroblast-specific protein 1 (FSP-1), N-cadherin, and vimentin [45]. Functionally, these transformed cells exhibit enhanced migratory capacity, increased secretion of ECM components, and active pro-fibrotic signaling, directly contributing to capillary rarefaction, microvascular dysfunction, and progressive renal interstitial fibrosis [46].

At the mechanistic level, EndMT in DKD is primarily driven by a complex interplay of fibrotic and inflammatory signals, centering on the TGF-β pathway. TGF-β induces EndMT through both Smad-dependent (Smad2/3) and non-Smad pathways (e.g., PI3K/AKT, MAPK, NF-κB), activating transcription factors like Snail, Slug, and Twist [47,48]. These factors drive the phenotypic switch by repressing endothelial gene programs and activating mesenchymal ones. Other pathways, including Notch, Wnt/β-catenin, and inflammatory cytokine signaling, also contribute to EndMT regulation, forming a tangled network that promotes endothelial dysfunction and fibrosis [46,49,50,51].

An increasing body of evidence indicates that EndMT is not a transient, reversible event, but can be durably maintained by “metabolic memory” through epigenetic mechanisms. Under chronic hyperglycemic stress, endothelial cells develop persistent epigenetic alterations, including DNA methylation, histone modifications, and ncRNA dysregulation. These modifications establish a stable, heritable transcriptional program that sustains the EndMT state long after glycemic normalization [52,53]. For example, hyperglycemia-induced histone modifications (such as H3K4 methylation) and DNA methylation changes can persistently activate the TGF-β signaling pathway and EndMT-related transcription factors. Similarly, ncRNAs like miRNAs and lncRNAs stabilize the EndMT phenotype by targeting key regulatory molecules. The persistent adoption of a mesenchymal fate by endothelial cells exemplifies the “legacy effect” of metabolic memory at the vascular level.

From a pathological perspective, EndMT not only increases the myofibroblast pool but also exacerbates peritubular capillary rarefaction and local hypoxia, which in turn amplifies pro-fibrotic signals, creating a self-perpetuating pathological cycle. Therefore, EndMT and EMT jointly form a critical cellular basis for renal fibrosis in DKD, and their sustained activation under metabolic memory-associated epigenetic regulation suggests that endothelial cells represent a highly promising, albeit less explored, therapeutic target.

### 2.4. Comprehensive Regulation Network and Positive Feedback Mechanism

In summary, a highly interconnected regulatory network has been constructed using multiple signaling pathways centered around TGF-β. There are extensive intersections between pathways such as Smad, MAPK, PI3K/AKT, Notch, Wnt/β-catenin, and Hippo-YAP/TAZ, creating multiple positive feedback loops that continuously amplify pro-fibrotic signals. As summarized in (Figure 1), this network not only drives the occurrence of EMT and EndMT, but more importantly, maintains their sustained activation, ultimately leading to the continuous deposition of ECM and irreversible progression of renal fibrosis.

Moreover, this “self-amplifying” signal network provides a mechanistic explanation for the continued progression of fibrosis even after the initial stimulus is eliminated, and may constitute an important molecular basis for the “metabolic memory” effect.

## 3. Metabolic Memory-Mediated Epigenetic Regulation Sustains Pro-Fibrotic Signaling Pathways in DKD

Metabolic memory, also known as the legacy effect [54], is a key concept proposed based on milestone clinical trials such as DCCT/EDIC and UKPDS [55,56,57]. Its core definition is a kind of persistent “memory” formed by the body in response to early hyperglycemia exposure. Even if the subsequent blood glucose levels as well controlled, this memory can continue to drive the initiation and development of diabetes complications (such as DKD). The concept of “metabolic memory” has greatly deepened the understanding of the pathological mechanism of chronic complications of diabetes, and also promoted the exploration of its molecular basis in the academic community.

The pivotal question in DKD pathogenesis is how metabolic memory sustains the continuous activation of pro-fibrotic signaling networks at the molecular level. Memory is not a single, isolated event; rather, it establishes a stable, and often self-sustaining, regulatory layer through epigenetics that exerts long-term effects on signal transduction.

In the early hyperglycemic environment, kidney cells are exposed to metabolic stressors, such as AGEs, ROS, and chronic inflammatory stimuli [57,58]. These factors acutely activate key pathways like TGF-β/Smad, NF-κB, Wnt/β-catenin, and Notch. However, transient activation cannot explain the unrelenting progression of DKD after blood glucose control. Mounting evidence shows that early hyperglycemic stimulation induces stable epigenetic changes, including DNA methylation, histone modification, and aberrant ncRNA expression, that convert these “transient signals” into “long-term transcriptional programs” [59].

**Figure 2:**
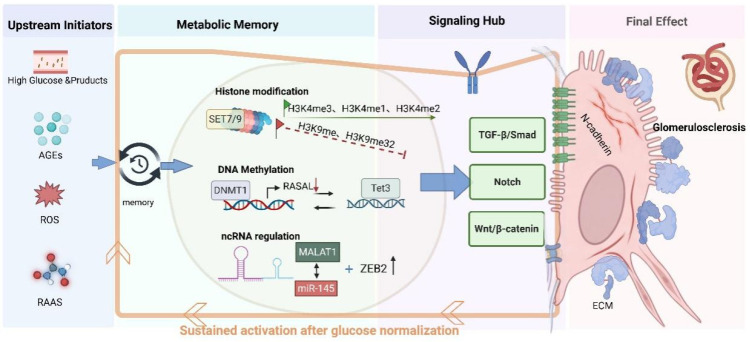
Hyperglycemia-induced factors (AGEs, ROS, RAAS) establish metabolic memory through epigenetic modifications, including histone modifications (e.g., SET7/9-mediated H3K4 methylation), DNA methylation (DNMT1, Tet3, RASAL1), and ncRNA regulation (MALAT1/miR-145/ZEB2 axis). These changes persist after glucose normalization and maintain activation of key signaling pathways (TGF-β/Smad, Notch, Wnt/β-catenin), ultimately promoting ECM accumulation and glomerulosclerosis. Green flag means activation marks; red flag means repressive marks; “↑” increase or activation; “↓” decrease or inhibition. (Created in BioRender. Ran, R. (2026) https://BioRender.com/ypkgu5t, accessed on 1 April 2026).

At the molecular level, epigenetic modifications act as a “memory storage system” that maintains the activated state of signaling pathways. For instance, H3K4 methylation and H3K27 acetylation in the promoter regions of fibrogenic genes persistently promote the transcriptional activity of TGF-β and its downstream targets [60,61]; DNA methylation changes in key regulatory genes enhance fibrogenic signaling or inhibit anti-fibrotic pathways. Moreover, ncRNAs (miRNAs, lncRNAs, and circRNAs) exert fine-tuning regulation on these pathways by targeting Smad proteins, β-catenin, and transcription factors such as Snail and Twist, further reinforcing their activated state [62]. These relationships are partially illustrated in Figure 2.

Crucially, these epigenetically maintained signaling pathways do not operate in isolation, but form multiple positive feedback networks through complex crosstalk. For example, TGF-β signaling induces the expression of DNA methyltransferases (DNMTs) and histone-modifying enzymes, which in turn enhance TGF-β pathway activity. Simultaneously, interactions with Wnt/β-catenin, Notch, and inflammatory pathways further amplify the pro-fibrotic response and stabilize the mesenchymal transition program. This integrated system creates a cellular state where kidney cells are “primed” and hyper-responsive to fibrotic stimuli, even after glucose normalization [63]. The continuous activation of this signaling network drives the long-term maintenance of EMT and EndMT, leading to persistent ECM deposition and promoting renal fibrosis.

In summary, metabolic memory bridges “early metabolic damage” and “long-term signal dysregulation” through epigenetic mechanisms, providing a unified explanation for DKD’s chronic progression and firmly establishing the epigenetic regulation of signaling pathways as a crucial therapeutic target.

## 4. Epigenetic Mechanisms Drive Mesenchymal Transition in DKD

The concept of epigenetics was first proposed by Waddington in 1942 [64], and its core meaning is to achieve heritable regulation of gene expression without altering the DNA sequence. This feature provides an important theoretical basis for explaining the persistent pathological damage and “metabolic memory” phenomenon in chronic diseases such as DKD.

In the pathological condition of DKD, epigenetic regulatory networks represented by histone modification, DNA methylation, and ncRNA reshape transcriptional regulatory programs by targeting the promoter or enhancer regions of EMT-related genes, thereby continuously activating EMT and related pro-fibrotic signaling pathways, ultimately promoting the progression of renal fibrosis [63].

### 4.1. Histone Modification Drives Mesenchymal Transition

Histones are alkaline proteins mainly present in the chromatin of eukaryotic cells, which together with DNA form nucleosome structures. The N-terminal tails of histones are subject to a variety of dynamic post-translational modifications mediated by specific enzyme systems. For example, histone acetylation is catalyzed by histone acetyltransferases (HATs) and removed by histone deacetylases (HDACs); histone methylation is regulated by histone methyltransferases (HMTs) and demethylases (HDMs); phosphorylation is mediated by protein kinases and reversed by phosphatases; ubiquitination is catalyzed by E3 ubiquitin ligases and removed by deubiquitinating enzymes (DUBs) [65,66,67,68,69]; and emerging modifications such as histone lactylation are also dynamically regulated [70]. These modifications mainly occur on specific amino acid residues, including lysine acetylation, lysine and arginine methylation, serine/threonine phosphorylation, and ubiquitination, contributing to the regulation of chromatin structure and gene expression. Among them, histone acetylation and histone methylation are the main types of histone modifications that drive renal interstitial fibrosis under high-glucose conditions. They dynamically regulate chromatin status and precisely regulate the expression of pro-fibrotic genes [62,71,72].

#### 4.1.1. The Regulatory Role of Histone Acetylation

Histone acetylation is catalyzed by histone acetyltransferases (HATs, such as p300, CBP) [73], which add acetyl groups to histone lysine residues to neutralize their positive charge, contributing to a weakened interaction between histones and negatively charged DNA. This altered histone–DNA interaction promotes gene transcription by keeping chromatin in an open state. In contrast, the reverse process (deacetylation) is mediated by histone deacetylases (such as HDAC1/2, Sirtuins) [74], which condense chromatin and inhibit target gene expression. The imbalance between histone acetylation and deacetylation is a key mechanism driving EMT and renal fibrosis in DKD.

It has been reported that histone acetylation modification is involved in regulating the expression of ECM-related genes in DKD. In a high-fat diet (HFD) combined with streptozotocin (STZ)-induced DKD mouse model, transcription cofactor MRTF-A can recruit the key component WD repeat-containing protein 5 (WDR5) of the HAT family p300 and histone H3K4 methyltransferase complex to bind to the collagen I promoter. By increasing the activation of histone modifications such as H3K18/27ac and H3K4me3, the transcriptional activity of type I collagen genes is significantly enhanced, ultimately leading to extensive ECM deposition and worsening of renal fibrosis [75]. In addition, the expression of pro-fibrotic genes such as PAI-1 (inhibiting ECM degradation), p21 (inducing cell cycle arrest and mesangial cell hypertrophy), CTGF, and others are also precisely regulated by histone modifications [76,77,78,79]. Histone deacetylases are divided into four categories: Class I (HDAC1, 2, 3, 8), Class II (HDAC4, 5, 6, 7, 9, 10), Class III (sirtuins, SIRT1-7) and Class IV (HDAC11) [80], in which Class I and II HDACs play an important role in kidney damage caused by diabetes [81,82]. In STZ-induced DKD mouse and high-glucose/palmitic acid (HG/Pal)-treated renal tubular epithelial cells (HK-2 cells), HDAC2 activity was significantly increased. HDAC2 can directly bind to the promoter region of bone morphogenetic protein 7 (BMP-7) and inhibit BMP-7 transcription through deacetylation. As a member of the TGF-β superfamily, BMP-7 can antagonize TGF-β/Smad2/3- mediated EMT by activating the Smad1/5/8 signaling pathway, suppressing the fibrotic process [83]. Therefore, pathological activation of HDAC2 can alleviate its inhibition of EMT by inhibiting BMP-7 expression, ultimately exacerbating renal fibrosis. Additionally, the activation of AKT (phosphorylation sites Ser473 and Thr308) induced by high-glucose level improves the expression of HDAC5, leading to the promotion of EMT by upregulating TGF-β1. The inhibition of PI3K/AKT pathway due to PI3K inhibitor LY294002 treatment or AKT phosphorylation mutation can reduce the expression of HDAC5 and TGF-β1 in HK-2 cells cultured with high-glucose in vitro, thereby inhibiting EMT in renal tubular cells [84].

#### 4.1.2. The Regulatory Role of Histone Methylation

Histone methylation is a reversible modification regulated by histone lysine methyltransferases (HMTs) and demethylases (HDMs). The biological function of this modification strictly depends on the site where it occurs and the degree of methylation, thus forming a complex “histone code”. In general, activating histone markers (such as H3K4me3) promote gene transcription by constructing open chromatin structures, while inhibitory markers (such as H3K9me3, H3K27me3) induce chromatin condensation by recruiting inhibitory complexes such as heterochromatin, thereby silencing gene expression [85]. Representative examples are provided in Table 1.

In the process of DKD renal fibrosis, the dynamic imbalance of histone methylation regulatory systems (HMTs/HDMs) can directly disrupt the transcriptional regulatory network of pro-fibrotic genes. For example, histone lysine methyltransferase SET7/9 can be recruited to the promoter regions of pro-fibrotic genes such as Col1a1, CTGF, PAI-1, by increasing the levels of activated chromatin markers (such as H3K4me1, H3K4me2, and H3K4me3) and decreasing the levels of inhibitory markers (such as H3K9me2 and H3K9me3), thereby exacerbating fibrosis [79]. Similarly, in high-glucose (HG)-treated rat mesangial cells (MCs), a decrease in H3K9me2 is associated with an increase in H3K4me1/3 and SET7/9 occupancy on the p21 promoter, promoting p21 gene expression, inducing mesangial cell cycle arrest and hypertrophy, and thereby accelerating kidney damage [86]. In addition, in rat glomerular mesangial cells, hyperglycemia suppresses the H3K27me3 methyltransferase Ezh2 and activates H3K27me3 demethylases Jmjd3 and UTX, relieving transcriptional inhibition of TGF-β1 and maintaining its high expression [87].

### 4.2. DNA Methylation Drives Mesenchymal Transition

DNA methylation is a key process in epigenetic modification. In cytosine-guanine (CpG) dinucleotides, a methyl group is transferred from S-adenosylmethionine (SAM) to the 5th carbon atom of cytosine, forming 5-methylcytosine. Typically, a high level of methylation in gene promoter regions can inhibit transcription factor binding and silence gene expression, while a low level of methylation can enhance promoter activity and promote gene transcription [88,89]. In DKD, the persistent changes in DNA methylation induced by high glucose can affect the expression of EMT-related genes, which is an important molecular basis for metabolic memory.

#### 4.2.1. DNA Methylation Regulates Mesenchymal Transition

The hypermethylation of Ras GTPase-activating-like protein 1 (RASAL1) gene is a key event driving fibroblast activation in the process of renal fibrosis [89]. Under normal circumstances, RASAL1 can inhibit the activation of the Ras/MAPK pathway by enhancing the hydrolytic activity of Ras GTPase; in a high-glucose environment, the methylation of CpG islands in the RASAL1 promoter region mediated by methyltransferase DNMT1 leads to silencing of RASAL1 expression. The absence of RASAL1 will release the inhibition of the Ras/MAPK signaling pathway, leading to excessive activation of Ras, ultimately promoting fibroblast activation and massive secretion of ECM. In addition, TGF-β1 can further induce the expression of DNMT1, enhance the methylation of the RASAL1 promoter, forming a positive feedback loop of “TGF-β1-DNMT1-RASAL1-Ras/MAPK”, and continuously amplify pro-fibrotic signals [90].

Regarding the pathological mechanism mentioned above, BMP-7, as a member of the TGF-β superfamily with anti-fibrotic properties, exhibits significant protective activity against renal fibrosis. BMP-7 was demonstrated to significantly upregulate the mRNA and protein expression of the DNA demethylase TET3 and enhance its catalytic activity in STZ-induced DKD mouse models and kidney biopsy tissues from patients with DKD. Tet3 can specifically recognize 5-methylcytosine (5mC) in the RASAL1 promoter region and gradually oxidize it to 5-hydroxymethylcytosine (5hmC); 5hmC can not only directly reduce the inhibitory effect of methylation in the promoter region, but also further transform into unmethylated cytosine through the base excision repair mechanism, effectively reversing the high methylation state of RASAL1 gene and restoring its expression. The re-expression of RASAL1 protein can effectively inhibit the overactivation of the Ras/MAPK pathway, ultimately achieving the reversal of renal fibrosis [91].

#### 4.2.2. DNA Methylation Regulates TGF-β1 Expression

TGF-β1, as the core inducer of EMT, is also regulated by DNA methylation. For example, in the glomerular mesangial cells of db/db diabetic mice, the binding site for transcription factor USF1 in the TGF-β1 promoter region was significantly demethylated, and this change enhanced the binding ability of USF1 to the promoter. At the same time, with the decreased DNMT1 binding, the TGF-β1 transcriptional activity eventually increased significantly [92]. The high expression of TGF-β1 can continuously drive EMT and exacerbate glomerulosclerosis and interstitial fibrosis by activating the Smad pathway and non-canonical pathway.

#### 4.2.3. Crosstalk Between DNA Methylation and Other Epigenetic Mechanisms

DNA methylation often interacts with other epigenetic mechanisms to jointly regulate renal fibrosis. For example, Klotho is an anti-aging and anti-fibrotic protein, and its downregulation is an important manifestation of DKD renal fibrosis. In a mouse model of renal fibrosis induced by unilateral ureteral obstruction (UUO), TGF-β1 can indirectly upregulate the levels of DNMT1 and DNMT3a (a de novo DNA methyltransferase) by inhibiting the expression of miR-152 and miR-30a; DNMT1/3a can bind to the Klotho promoter region, leading to hypermethylation and silencing of Klotho expression. The absence of Klotho will release its inhibition of the TGF-β/Smad pathway, further exacerbating EMT and fibrosis, forming a vicious cycle of “TGF-β1-miR-152/30a-DNMT1/3a-Klotho” [93]. In addition, genistein can restore Klotho expression through a dual mechanism: on one hand, it inhibits DNMT1/3a activity, and reduces Klotho promoter methylation levels; on the other hand, it inhibits histone deacetylase activity, increases Klotho promoter region histone acetylation levels, and ultimately alleviates renal fibrosis by upregulating Klotho expression [94]. Representative examples are provided in Table 2.

### 4.3. Non-Coding RNA (ncRNA) Drives Mesenchymal Transition

Non-coding RNAs (ncRNAs) are RNA molecules that do not encode proteins, mainly including miRNAs, lncRNAs, and circRNAs. In DKD, these three groups of ncRNAs participate in the pathological process of EMT by directly targeting the core molecules of mesenchymal transition or indirectly regulating the upstream signaling pathways [63]. Their differential expression is often persistent and is an important manifestation of metabolic memory at the epigenetic level.

#### 4.3.1. The Regulatory Role of miRNAs

miRNAs regulate gene expression by binding to the 3′ untranslated region (3′UTR) of target mRNAs, inhibiting mRNA translation or promoting its degradation. Beyond their intracellular function, miRNAs can also be released by cells and transported to adjacent or distant cells through exosomes, small extracellular vesicles (sEVs), or binding to plasma proteins, producing paracrine effects and mediating intercellular communication [95]. These miRNAs play dual roles in the pathological mechanisms of DKD, promoting renal fibrosis by enhancing the TGF-β signaling pathway, and conversely contributing to the maintenance of epithelial phenotype or prevention of ECM synthesis and deposition. Representative examples are provided in Table 3.

In DKD renal fibrosis, multiple miRNAs play a role by regulating EMT-related genes. The physiological function of miR-21 is closely related to EMT, and its overexpression enhances TGF-β1-induced EMT by directly targeting Smad7 and indirectly downregulating Smad3 [96,97]. For example, high glucose can induce HK-2 cells to secrete sEVs rich in miR-21-5p. After these sEVs are taken up by adjacent renal tubular epithelial cells, miR-21-5p can promote cell proliferation, migration, and EMT by targeting and inhibiting the expression of BTG2 (B cell translocation gene 2, a tumor suppressor gene); knocking down miR-21-5p can significantly reverse the above effects, indicating that miR-21-5p is a key promoter of EMT in DKD [98].

Wang et al. found that miR-30b-5p was downregulated not only in db/db mice and HK-2 cells incubated with high glucose, but also in human DKD kidney tissue. At the same time, in db/db mice, the expression of miR-30b-5p was negatively correlated with the expression level of Snail1, confirming that miR-30b-5p delayed EMT in HK-2 cells by targeting and inhibiting Snail1 mRNA in DKD kidney tissue [99]. In addition, studies have reported that miR-30c targets Snail1 to inhibit TGF-β1-mediated EMT in renal tubular epithelial cells. It has also been found that miR-30c synergizes with miR-26a to enhance the inhibition of TGF-β1-mediated EMT, suggesting that the miR-30 family may be a potential protective factor for DKD [100].

#### 4.3.2. The Regulatory Role of lncRNAs

Although lncRNAs do not encode proteins, they can regulate gene expression through various mechanisms. Some lncRNAs can directly bind to DNA to regulate transcription, act as competitive endogenous RNAs (ceRNAs) to bind to miRNAs, and interact with proteins to affect their localization or activity. In DKD, lncRNAs regulate the expression level of EMT-related genes through the aforementioned mechanisms, with ceRNA mechanism and nuclear transcriptional regulation being the most important modes of action [101,102]. Representative examples are provided in Table 3.

The ceRNA hypothesis was proposed by the Pandolfi research group at Harvard Medical School in 2011, which suggests that lncRNAs can competitively bind to miRNAs to release their inhibition of target mRNA, thereby upregulating the expression of target genes. Research has shown that MALAT1 expression is significantly upregulated in HK-2 cells induced by high glucose; MALAT1 can act as a ceRNA that competes with miR-145 to bind to the mRNA of the target gene ZEB2 (key transcription factor for EMT), inhibiting miR-145’s degradation of ZEB2 and leading to increased expression of ZEB2, thereby promoting EMT in renal tubular epithelial cells [103]. In addition, studies have shown that MALAT1 can further exacerbate EMT and renal fibrosis by activating the Wnt/β-catenin pathway [104]. Wang et al. found that GAS5 expression was upregulated in DKD mouse models and HK-2 cells treated with TGF-β1; GAS5 can act as a ceRNA binding miR-96-5p, relieving the inhibition of miR-96-5p on the pro-fibrotic protein fibronectin 1 (FN1), leading to an increase in FN1 expression and promoting ECM deposition and EMT [105].

Some nuclear-localized lncRNAs regulate gene transcription by interacting with transcription factors or their cofactors. A representative example is lincRNA-p21, which functions as a transcriptional coactivator by binding to hnRNP-K, facilitating its recruitment to the p21 promoter and enhancing p53 binding, thereby promoting p53-dependent transcription in cis [106].

In addition, cytosolic lncRNAs can regulate mRNA translation and protein stability. LncRNA KIFAP3-5:1 is downregulated in renal tubular epithelial cells treated with high glucose. Indeed, the overexpression of lncRNA KIFAP3-5:1 is found to reduce EMT and renal fibrosis by directly binding to mRNA of PRRX1 (pairing related homologous box 1, a pro-EMT transcription factor), inhibiting its expression. Furthermore, the study found that the expression level of lncRNA KIFAP3-5:1 in plasma was positively correlated with eGFR levels, and the expression level of lncRNA KIFAP3-5:1 in plasma of G3 stage DKD patients was significantly lower than that of the control group, suggesting that it can be a potential biomarker for the diagnosis and prognosis evaluation of DKD [107].

#### 4.3.3. The Regulatory Role of circRNAs

CircRNAs are a class of ncRNAs with closed circular structures that are more stable than linear RNAs due to the absence of a free 5′ cap and 3′ polyadenylation tail. They mainly participate in gene expression regulation through interactions with proteins or direct transcriptional regulation [108]. At present, although research on circRNAs in DKD renal fibrosis started relatively late, there has been a significant increase in relevant evidence in recent years. Their regulatory roles in renal intrinsic cells such as mesangial cells, tubular epithelial cells, and podocytes have gradually become clear.

##### The Regulatory Role of circRNAs in Glomerular Mesangial Cells

Abnormal proliferation of MCs and excessive secretion of ECM are the core pathological features of DKD glomerulosclerosis. Studies have found that high-glucose stimulation can significantly upregulate the expression of circRNA 15698, circLRP6, circ-0080425, etc., in glomerular mesangial cells. Among them, circRNA-15698 sponges miR-185 to release its inhibition of the target gene TGF-β1 (fibrosis core regulatory factor), promoting the synthesis of ECM components such as Col1a1 and FN1 [109]; circ-LRP6 activates the TLR4/NF-κB pathway by sponging miR-205, while promoting MC proliferation, oxidative stress, release of inflammatory factors (IL-6, TNF-α), and accumulation of ECM [93]; circ-0080425, circ-0000491, circ-0123996, and circ-00037128 promote MC proliferation and fibrosis at different levels through the miR-24-3p/FGF11, miR-101b/TGF-β RI, miR-149-5p/Bach1, and miR-17-3p/AKT3 axes, respectively [110,111,112,113].

In addition to cell model studies, clinical sample validation further revealed the disease-associated value of circRNAs. A clinical study involving 10 healthy controls (CT group), 10 patients with simple diabetes (DM group) and 10 patients with diabetic kidney disease (DKD group) showed that the serum circ-0054633 level increased gradually in the three groups (CT group < DM group < DKD group), and its expression level was significantly positively correlated with the key clinical indicators of DKD patients—urinary albumin creatinine ratio (ACR) and the degree of renal fibrosis, suggesting that circ-0054633 may be a potential serum marker for DKD assessment. Functional experiments further confirmed the pro-fibrotic effect of circ-0054633: in a human renal interstitial cell model exposed to high-glucose stress in vitro, silencing circ-0054633 expression through small interfering RNA (siRNA) can significantly inhibit abnormal cell proliferation and reduce the accumulation of ECM components such as Col1a1 and FN1; in vivo experiments, silencing circ-0054633 in the kidney tissue of db/db diabetic mice through targeted delivery technology can also effectively reduce the degree of glomerulosclerosis and renal interstitial fibrosis. Mechanistic studies have shown that circ-0054633 exerts a “molecular sponge” effect by sequestering miR-136-5p; silencing circ-0054633 can release free miR-136-5p, thereby downregulating the expression of its target gene Smad3 (the core effector molecule of the TGF-β/Smad pathway), ultimately inhibiting the activation of the fibrosis signaling pathway [114].

Contrary to the pro-fibrotic molecules mentioned above, circ-AKT3 and circ-LARP4 were significantly downregulated in high-glucose-cultured MCs. Circ-AKT3 maintains epithelial phenotype and inhibits ECM deposition by regulating the miR-296-3p/E-cadherin signaling pathway [115]. It is worth noting that this regulatory axis also plays an inhibitory role in the metastasis of renal cell carcinoma, suggesting that the circRNA-mediated regulatory network may have disease universality [116]. Circ-LARP4 inhibits MCs proliferation and fibrosis by sponging miR-424, while promoting cell apoptosis and maintaining cellular homeostasis [117].

In addition, the study found that different circRNAs can synergistically regulate the function of MCs by targeting the same miRNA. For example, miR-143 can be sponged by both circ_DLGAP4 and circ_0000064, and elevated expression of both can promote MCs proliferation and fibrosis [118,119]; miR-185-5p can be targeted separately by circ-WBSCR17 in TECs and circHIPK3 in MCs, jointly inhibiting the activity of this miRNA in both tubulointerstitial and glomerular regions, and synergistically promoting renal fibrosis [119,120].

##### The Regulatory Role of circRNAs in Renal Tubular Epithelial Cells (TECs)

The EMT and injury of TECs are a key process in DKD renal interstitial fibrosis. High-glucose environment can induce upregulation of circRNAs such as hsa-circ-0003928, circ-WBSCR17, circACTR2, and circEIF4G2 in TECs. Hsa-circ-0003928 can upregulate the expression of target gene Anxa2 (membrane associated protein A2) by sponging miR-151-3p, promote the secretion of inflammatory factors such as IL-6 and MCP-1 by TECs, inhibit cell apoptosis, and exacerbate renal tubular injury [121]; circ-WBSCR17 targets miR-185-5p, relieving its inhibition of SOX6 (pro-fibrotic transcription factor) and enhancing the inflammatory response and fibrosis of TECs [120]; circACTR2 can significantly promote high-glucose-induced apoptosis, inflammation, and fibrosis of TECs, but its specific regulatory mechanism still needs further validation [122]; circEIF4G2 upregulates the synthesis of fibrosis-related proteins (α-SMA, Col1a1) through the miR-218/SERBP1 pathway, accelerating renal interstitial fibrosis [123].

It is worth noting that the same circRNA may exert heterogeneous effects by targeting different miRNAs in different cell types. For example, circHIPK3 promotes fibrosis in MCs by inhibiting miR-185-5p [119], but in TECs it can reduce high-glucose-induced cytotoxicity and exhibit protective effects against cytotoxicity by sponging miR-326/miR-487a-3p [124], suggesting that the function of circRNAs is cell-specific and needs to be analyzed in conjunction with specific cellular microenvironments.

##### The Regulatory Role of circRNAs in Podocytes

Podocyte injury is the primary cause of abnormal glomerular filtration barrier function in the early stage of DKD. It has been revealed that circ-0000285 is significantly upregulated both in podocytes treated with high glucose and in kidney tissue of DKD mice. As a result, MAPK6 signaling pathway is activated due to circ-0000285-mediated miR-654-3p sponging, inducing podocyte apoptosis and downregulating the expression of nephrin and podocin, ultimately leading to proteinuria and glomerular injury [125], which provides a new target for the treatment of early podocyte lesions in DKD.

At present, the majority of studies on circRNAs are based on experiments in vitro, while functional validation in vivo remains limited. It should be highlighted that ultrasound microbubble-mediated gene transfection technology was conducted to specifically deliver circRNA-010383 expression plasmid to the kidney of diabetes-affected mice, and intermittent ultrasound intervention was demonstrated to restore the expression level of circRNA-010383 in mouse kidney tissue, contributing to a significant reduction in renal interstitial fibrosis [126]. This study not only confirms the anti-fibrotic effect of circRNAs in vivo, but also provides a technical solution for kidney-specific delivery of circRNAs, laying the foundation for subsequent clinical translation.

**Table 3:** Regulatory mechanisms of non-coding RNAs in renal fibrosis of DKD.

Modification Mechanism	Experimental Object	Cofactors/Targets	Expression Change	Effect	Reference
miRNA	High-glucose-stimulated HK-2 cells	BTG2	miR-21-5p ↑; BTG2 ↓	EMT ↑; Fibrosis ↑	[98]
miRNA	db/db mice, High-glucose HK-2 cells, Human DN renal tissue	Snail1	miR-30b-5p ↑; Snail1 ↓	EMT ↓; Fibrosis ↓	[99,100]
lncRNA (ceRNA mechanism)	High-glucose-induced HK-2 cells	MALAT1(lncRNA), miR-145, ZEB2	MALAT1 ↑; miR-145 ↓; ZEB2 ↑	EMT ↑; Fibrosis ↑	[103]
lncRNA (ceRNA mechanism)	DKD mouse model, TGF-β1-induced HK-2 cells	GAS5(lncRNA), miR-96-5p, FN1	GAS5 ↑; miR-96-5p ↓; FN1 ↑	EMT ↑; Fibrosis ↑	[105]
lncRNA (Transcriptional regulation)	HG-treated renal tubular epithelial cells; Plasma from DKD patients	lnc-KIFAP3-5:1, PRRX1	lnc-KIFAP3-5:1 ↑; PRRX1 ↓	EMT ↓; Fibrosis ↓	[107]
circRNA (Molecular sponge)	High-glucose-stimulated mesangial cells	circRNA-15698, miR-185;	-	ECM(Col1a1, FN1) ↑	[109]
circRNA (Molecular sponge):	db/db mice; HG-stressed human renal interstitial cells	circ-0054633, miR-136-5p, Smad3	circ-0054633 ↓; miR-136-5p ↑; Smad3 ↓	Fibrosis ↓	[114]
circRNA (Molecular sponge)	High-glucose-cultured mesangial cells	circ-AKT3, miR-296-3p	circ-AKT3 ↓; miR-296-3p ↓	ECM ↓; Fibrosis ↓	[115]
circRNA (Molecular sponge)	Tubular epithelial cells (TECs) under high-glucose conditions	Has-circ-0003928, miR-151-3p, Anxa2	Anxa2 ↑	tubular injury ↑	[121]
circRNA (Molecular sponge)	Tubular epithelial cells (TECs) under high-glucose condition	circ-WBSCR17, miR-185-5p, SOX6	SOX6 ↑	Fibrosis ↓	[120]

Table Notes: “↑” increase or activation; “↓” decrease or inhibition; “-” the information was not explicitly specified in the text.

### 4.4. The Cross-Interaction Between Epigenetic Regulation

Based on existing studies, “metabolic memory” continuously drives EMT through a multi-level epigenetic regulation network, promoting renal fibrosis progression in DKD. Early hyperglycemic environments can induce DNA methylation, histone modifications, and changes in ncRNA (such as miRNAs) expression profiles, leading to the formation of relatively stable epigenetic “imprints”. Consequently, even if the hyperglycemia is reversed, these epigenetic changes maintain the sustained activation of EMT-related transcription factors by regulating key signaling pathways such as TGF-β.

Furthermore, different epigenetic regulatory mechanisms do not act independently, but form an intricate network through complex cross-regulation. For example, DNA methylation can indirectly affect EMT by regulating the expression of miRNAs, which in turn can target DNMTs or histone-modifying enzymes in reverse, constructing feedback regulatory loops. Meanwhile, histone modification and DNA methylation synergize in chromatin remodeling, jointly determining the transcriptional activity of genes. This multi-level and interactive epigenetic regulatory system not only amplifies and stabilizes EMT signaling, but may also constitute a key molecular basis for the long-term maintenance of “metabolic memory” in DKD, thereby driving the sustained progression of renal fibrosis.

## 5. The Application Prospects of Epigenetic Biomarkers in DKD

Epigenetic biomarkers have shown great potential in the diagnosis, prognosis evaluation, and treatment response monitoring of DKD due to their high stability and convenient detection (which can be detected from body fluids such as blood and urine). Currently, multiple studies have confirmed that some epigenetic molecules can serve as potential biomarkers for DKD, providing a theoretical basis for clinical translation.

### 5.1. miRNAs as Biomarkers

miRNAs have been proven to have clear biomarker value in DKD and related chronic kidney disease (CKD) due to their high stability (mediated by extracellular vesicles or protein binding in body fluids) and mature detection methods (such as qPCR and RNA sequencing). In the aged CKD mouse model, the expression of miR-146a in renal tissue was significantly increased and positively correlated with the degree of infiltration of renal interstitial inflammatory cells. Additionally, the study demonstrated that the level of miR-16a in the urine sediment of CKD mice was significantly higher than that in the supernatant, and the sequence of human miR-16a was the same as that of rodents [127], suggesting that miR-16a may become a biomarker for the diagnosis and evaluation of inflammatory activity in CKD (including DKD). Clinical studies have found that the level of miR-21 in the serum of DKD patients is significantly higher than that in healthy individuals and is positively correlated with key clinical indicators of DKD, including albumin-to-creatinine ratio (ACR) and serum creatinine, and negatively correlated with eGFR [128]. Multivariate analysis further confirmed that serum miR-21 is an independent risk factor for DKD, suggesting that it can serve as a biomarker for the auxiliary diagnosis and severity assessment of DKD [128,129]. In addition, miR-30c is significantly downregulated in renal tissue and urine exosomes of DKD patients, and its expression level is negatively correlated with the degree of renal interstitial fibrosis [130]. Functional experiments have also found that overexpression of miR-30c can alleviate EMT progression by inhibiting Snail1 expression, suggesting that it is not only a prognostic marker for the risk of progression of DKD, but also a potential therapeutic target [131].

### 5.2. LncRNAs as Biomarkers

Although lncRNAs are more difficult to detect than miRNAs, their tissue specificity is stronger [132]. Abnormal expression of some lncRNAs is also highly correlated with human diseases, especially chronic diseases [133]. It has been reported that lncRNAs such as MALAT1, PVT1, and H19 can regulate the expression of ECM proteins and pro-fibrotic factors in DKD. For example, MALAT1, as a ceRNA of miR-145, can release the inhibition of target gene ZEB2 expression by miR-145, thereby inducing EMT and fibrosis; PVT1 (plasma cell tumor variant translocation 1) is commonly believed to play a carcinogenic role in cancer [134]. Studies have also found that knocking down PVT1 can inhibit cell proliferation and EMT [135,136]. Lowering PVT1 expression inhibits the expression of two key regulatory factors of ECM protein, TGF-β1 and plasminogen activator inhibitor-1 (PAI-1), which is the main inhibitor of ECM degradation [137,138]. On one hand, H19 can act as a ceRNA for miR-29a, blocking its expression and significantly increasing the activity of the TGF-β/Smad3 pathway [139]. On the other hand, it can also act as an endogenous RNA to reduce miR-17 levels and promote the production of α-SMA, Collagen Type IV (Col. IV), and Collagen Type I (Col. I) [140].

## 6. Therapeutic Targets and Intervention Strategies Based on Epigenetic Mechanisms

As the key role of epigenetic mechanisms in regulating EMT in DKD renal fibrosis is gradually elucidated, intervention strategies targeting relevant epigenetic regulatory factors are becoming a potential therapeutic approach. Unlike the traditional treatment mode that focuses on blood glucose control, epigenetic-targeted therapy is expected to exert potential anti-fibrotic effects by regulating abnormal transcription programs and intervening with the “metabolic memory” effect.

### 6.1. Epigenetic Enzymes as Therapeutic Targets

Epigenetic modifying enzymes are core regulators of chromatin status and gene expression, including histone-modifying enzymes and DNA methylation-related enzymes. Their abnormal activation plays a key role in DKD renal fibrosis.

HDACs are significantly upregulated in hyperglycemic environments, exacerbating EMT and ECM deposition by inhibiting the expression of anti-fibrotic genes (such as BMP-7) and promoting the activation of the TGF-β signaling pathway. Research has shown that HDAC inhibitors can alleviate renal fibrosis by restoring chromatin openness and inhibiting transcription of pro-fibrotic genes [141].

In addition, abnormal DNA hypermethylation mediated by DNMTs is widely present in DKD, for instance, by silencing anti-fibrotic genes such as RASAL1 and continuously activating the Ras/MAPK signaling pathway. Targeting DNMTs or promoting DNA demethylation has been shown to reverse some fibrotic phenotypes, suggesting their feasibility as potential therapeutic targets [142].

### 6.2. Non-Coding RNA-Targeted Therapy Strategy

NcRNAs, including miRNAs, lncRNAs, and circRNAs, play a key role in regulating EMT and renal fibrosis, and their abnormal expression is persistent, making them important molecular carriers of “metabolic memory”.

miRNAs can exert bidirectional regulation by targeting EMT-related transcription factors or signaling pathways. For example, inhibiting miR-21 or restoring miR-30 family expression can effectively alleviate the EMT process and reduce ECM deposition. In addition, lncRNAs such as MALAT1 and GAS5 regulate the expression of key pro-fibrotic genes through the ceRNA mechanism, and their targeted interventions have also shown potential anti-fibrotic effects.

In terms of treatment strategies, small interfering RNA (siRNA) and antisense oligonucleotides (ASOs) are currently the most widely studied ncRNA targeting technologies. SiRNA can mediate target RNA degradation through the RNA-induced silencing complex (RISC), while ASOs achieve specific inhibition of nuclear RNA by recruiting RNase H. For example, siRNA targeting Smad4 has been shown to significantly inhibit the progression of renal fibrosis, while ASOs targeting CHOP can improve glomerular and tubular injury [132,143,144].

### 6.3. Targeted Interventions for EMT and Key Signaling Pathways

As the core pathological process of DKD renal fibrosis, targeting upstream signaling pathways of EMT also has important therapeutic significance. Among them, the TGF-β signaling pathway, as the core driving factor of EMT, is currently the most extensively studied intervention target.

In addition, signaling pathways such as Notch, Wnt/β-catenin, and Hippo-YAP/TAZ form a complex cross-regulatory network with TGF-β, collectively maintaining the sustained activation of EMT. Intervention strategies targeting these signaling pathways are expected to suppress the amplification of pro-fibrotic signals at the network level, thereby more effectively blocking the progression of renal fibrosis.

### 6.4. Targeted Delivery and Clinical Translation Challenges

Although epigenetic-targeted therapy has shown promising prospects in basic research, its clinical translation still faces multiple challenges. Notably, due to the global characteristics of epigenetic regulation, related inhibitors may induce off-target effects and systemic toxicity. In addition, there are still limitations in the stability and delivery efficiency of nucleic acid drugs such as siRNA and ASOs in vivo [145,146].

Recently, research based on nanocarriers and kidney-specific delivery systems has provided new ideas for solving the above problems. In addition, epigenetic editing techniques, such as precise regulation strategies based on dCas9, are expected to achieve targeted regulation of specific gene loci, thereby improving therapeutic efficacy while reducing side effects.

## 7. Discussion

The persistent progression of DKD, even after strict glycemic control, presents a major clinical challenge, a phenomenon termed “metabolic memory.” This review systematically demonstrates that metabolic memory maintains the sustained activation of EMT and EndMT through epigenetic mechanisms, thereby constituting a key driver of renal fibrosis. Unlike transient signaling, epigenetic modifications transform early metabolic stress signals into stable, long-term pro-fibrotic gene expression programs. This leads to the phenotypic reprogramming of kidney cells and irreversible ECM deposition, underpinning the chronic nature of DKD.

Recent research has expanded the understanding of metabolic memory’s nature, revealing that it originates not only from hyperglycemia-induced oxidative stress but also involves changes in intracellular energy metabolism. For example, lactate accumulation from enhanced glycolysis is not merely a metabolic byproduct but a key epigenetic substrate. The newly discovered histone lactylation modifications (e.g., H3K14la) indicate that metabolic intermediates can directly enter the nucleus to regulate chromatin openness, upregulating EMT-related transcription factors like KLF5 and maintaining their activation [147,148,149]. This cascade from “metabolic substrates” to “gene regulation” provides a deeper molecular mechanistic framework for metabolic memory than traditional oxidative stress theory.

Notably, the role of EMT in DKD remains somewhat controversial. Increasing evidence from single-cell sequencing and spatial transcriptomics suggests that kidney cells may undergo a “partial EMT” state during fibrosis, retaining some epithelial features while acquiring pro-fibrotic functions. This high heterogeneity of renal cell populations indicates that epigenetic regulation likely plays a central role in maintaining this “pro-fibrotic activation state.” However, the differential response mechanisms of distinct cell subpopulations to metabolic memory require systematic elucidation. Future research should focus on identifying cell-specific epigenetic signatures and exploring why certain proximal tubular segments (e.g., the S3 segment) are more sensitive to metabolic memory, aiming for precise, cell-level interventions.

From a clinical translation perspective, intervention strategies targeting epigenetic regulation are promising but face significant hurdles. Although HDAC inhibitors and DNMT inhibitors have shown anti-fibrotic effects in basic research, their off-target toxicity and lack of tissue specificity limit their clinical development. Future research should explore novel interventions based on targeted delivery systems, such as renal tubule-specific nanocarriers, and precision epigenome-editing techniques, like dCas9-based regulators [150], to achieve targeted modulation of key pathogenic epigenetic marks while minimizing global gene perturbations. The incorporation of EndMT as a dual target alongside EMT also represents a critical expansion of therapeutic strategy.

In summary, the role of metabolic memory in DKD renal fibrosis is a multi-level and multi-dimensional regulatory process. Future research must integrate the intrinsic connections between altered metabolic states, epigenetic regulation, and cell phenotype transformation to construct a systematic mechanistic framework. With advances in multi-omics technologies and precise regulatory tools, intervention strategies targeting metabolic memory-related epigenetic imprints are expected to provide a new theoretical basis and potential treatment pathways for delaying DKD progression and improving long-term outcomes.

Although this article focuses on DKD, the principle of metabolic memory sustaining EMT/EndMT activation through epigenetic mechanisms may have broader applicability, reflecting a common regulatory pattern in CKD progression. Renal fibrosis is a final common pathway in various CKD forms, including hypertensive renal damage and glomerulonephritis. In these diverse etiologies, pathways like TGF-β, NF-κB, and Wnt/β-catenin are persistently activated, and epigenetic mechanisms under non-diabetic conditions also maintain this activation. This suggests that transient injurious stimuli can induce lasting epigenetic “imprints” that perpetuate the fibrotic response, making “metabolic memory” in DKD a specific manifestation of a broader “injury memory.” Therefore, targeting the “epigenetic-signaling pathway” interaction network that drives sustained fibrosis is expected to become a unified therapeutic strategy across different CKD etiologies, not limited to diabetes-related renal damage.

## Figures and Tables

**Table 1:** Regulatory mechanisms of histone modifications in renal fibrosis of DKD.

Modification Mechanism	Experimental Object	Cofactors/Enzymes	Histone Modification Change	Effect	Reference
Acetylation	DKD Mouse (HFD/STZ-induced)	MRTF-A, p300, WDR5	H3K18/27ac ↑ H3K4me3 ↑	ECM ↑; Fibrosis ↑	[75]
Acetylation	-	PAI-1, p21, CTGF	-	Fibrosis ↑	[76,77,78,79]
Deacetylation	STZ-induced DKD model; HG/Pal-treated HK-2 cells	HDAC2	Deacetylation at BMP-7 promoter	ECM ↑; Fibrosis ↑	[83]
Deacetylation	High-glucose-treated HK-2 cells	HDAC5	HDAC5 ↑	ECM ↑; Fibrosis ↑	[84]

Table Notes: “↑” increase or activation; “-” the information was not explicitly specified in the text or is not a classic signaling pathway.

**Table 2:** Regulatory mechanisms of DNA methylation in renal fibrosis of DKD.

Modification Mechanism	Experimental Object	Cofactors/Enzymes	Key Changes	Effect	Reference
DNA Hypermethylation	High-glucose milieu; Renal fibroblasts	DNMT1	RASAL1 ↓	ECM ↑; Fibrosis ↑	[90,92]
DNA Demethylation	STZ-induced DKD mouse model; Human DKD renal biopsy tissue	BMP7, DNA demethylase Tet3	BMP7 ↑ Tet3 ↑	Fibrosis ↓	[91]
Crosstalk of DNA Hypermethylation and Histone Modification	UUO-induced renal fibrosis mouse model	miR-152/30a DNMT1/3a	miR-152/30a ↓ DNMT1/3a ↑ Klotho ↓	Fibrosis ↑	[93]

Table Notes: “↑” increase or activation; “↓” decrease or inhibition. RASAL1: Ras GTPase-Activating-Like Protein 1; BMP7: Bone Morphogenetic Protein 7; UUO: Unilateral Ureteral Obstruction; Klotho: An anti-aging and anti-fibrotic protein.

## Data Availability

No new data were generated or analyzed in this study. Data sharing is not applicable to this article.
